# Deep Learning with Taxonomic Loss for Plant Identification

**DOI:** 10.1155/2019/2015017

**Published:** 2019-11-21

**Authors:** Danzi Wu, Xue Han, Guan Wang, Yu Sun, Haiyan Zhang, Hongping Fu

**Affiliations:** ^1^School of Landscape Architecture, Beijing Forestry University, Beijing 100083, China; ^2^School of Information Science and Technology, Beijing Forestry University, Beijing 100083, China; ^3^School of Cyber Science and Technology, Beihang University, Beijing 100191, China

## Abstract

Plant identification is a fine-grained classification task which aims to identify the family, genus, and species according to plant appearance features. Inspired by the hierarchical structure of taxonomic tree, the taxonomic loss was proposed, which could encode the hierarchical relationships among multilevel labels into the deep learning objective function by simple group and sum operation. By training various neural networks on PlantCLEF 2015 and PlantCLEF 2017 datasets, the experimental results demonstrated that the proposed loss function was easy to implement and outperformed the most commonly adopted cross-entropy loss. Eight neural networks were trained, respectively, by two different loss functions on PlantCLEF 2015 dataset, and the models trained by taxonomic loss led to significant performance improvements. On PlantCLEF 2017 dataset with 10,000 species, the SENet-154 model trained by taxonomic loss achieved the accuracies of 84.07%, 79.97%, and 73.61% at family, genus and species levels, which improved those of model trained by cross-entropy loss by 2.23%, 1.34%, and 1.08%, respectively. The taxonomic loss could further facilitate the fine-grained classification task with hierarchical labels.

## 1. Introduction

As the main form of life on the earth, plant plays an indispensable role in the ecosystem, which ensures the sustainable development of human society. Plant identification is a crucial component of plant ecological research workflow, which is the foundation to protect and develop the plant diversity. As for the general public, identifying plant and learning its knowledge is also an interesting and necessary experience. Although there are several methods of identifying plant, including taxonomic keys, written description, specimen comparison, and image comparison, expert determination is usually necessary [1]. Besides, for the large quantity of plant species and the low readability of taxonomic information, taxonomic knowledge and species identification skills are restricted to a limited and reducing number of persons [2, 3]. So, even for experts with professional plant knowledge, it is not practical to identify all kinds of plant species by the manual identification methods, while for non-experts, it seems to be more infeasible.

Image-based automatic plant identification has emerged as a hot spot in the field of computer vision [4]. In contrast to the coarse-grained ImageNet [5] classification task, image-based plant identification is a fine-grained classification task which aims to distinguish the family, genus, and the most specific species. In the past, nearly all machine learning methods relied on hand-crafted visual features (e.g., leaf vein and petal shape) [6–9], while the manual process was time-consuming and the extracted features were possibly incomplete. Moreover, these methods suffered from poor generalization for large-scale plant identification in complex environment. Recently, progress in deep learning [10–14] has demonstrated its outstanding performance on automatic feature extraction through data-driven approaches. Many works have turned sight to the combination of plant identification and neural networks, which significantly boosted the accuracy for large-scale plant recognition [15–17].

So far, for plant classification task, the most commonly adopted method of deep learning training was one-hot encoding with cross-entropy loss, which only used one-level labels of the taxonomic tree, such as species, and ignored the strong intra-family/genus similarities. By this means, the hierarchical structures of taxonomic tree were totally neglected. The optimizer could only optimize the model according to species-level information independently, without the rich supervision information derived from taxonomic tree. The models of these methods predicted the most specific species directly [17–19], while human experts generally identify plants from coarse to fine by matching the family, genus, and species along the taxonomic tree progressively. In practice, it is also useful to identify the family/genus correctly even if the species prediction is wrong.

Inspired by the hierarchical structure of taxonomic tree, the taxonomic loss was proposed to encode the taxonomic tree into the objective function of deep learning training. Then, the training algorithm could optimize the model with more supervision information derived from the hierarchical labels. The proposed method was easy to implement, compatible with end-to-end training, and effectively improves the performance of plant classification models. In summary, two contributions of this paper are listed as follows:

1. The taxonomic loss encoded taxonomic tree into the objective function by simple group and sum operation, which was easy to implement and compatible with end-to-end training.
2. The taxonomic loss facilitated the training of various deep neural networks, which further increased plant identification accuracies at species, genus, and family levels.

## 2. Materials and Dataset

Two different editions of PlantCLEF datasets (PlantCLEF 2015 [20] and 2017 [21]) were used to evaluate the performance of the proposed method, the images from which were collected from different locations by distinct contributors. Each image belongs to one of seven content-types (e.g., flower, fruit, and stem) and was annotated with hierarchical family, genus, and species labels according to the taxonomic tree, which organized the plant hierarchically in a coarse-to-fine fashion. So, the PlantCLEF datasets were suitable for evaluating the proposed algorithm on three-level granularities along the taxonomic tree.

The PlantCLEF 2015 dataset contains 113,205 images of 1,000 species and was divided into training set and testing set by the contest host. The training set of PlantCLEF 2017 consisted of two subsets: “trusted” and “noisy” set. Since this paper focused on the supervised training with ground truth, only “trusted” set was used for experiments, which contains 256,287 images with 10,000 species. And one-tenth samples from each individual species were selected randomly into the testing set.  Table 1 shows the details of the datasets used in this paper.

## 3. Taxonomic Loss for Deep Learning

**Figure 1:**
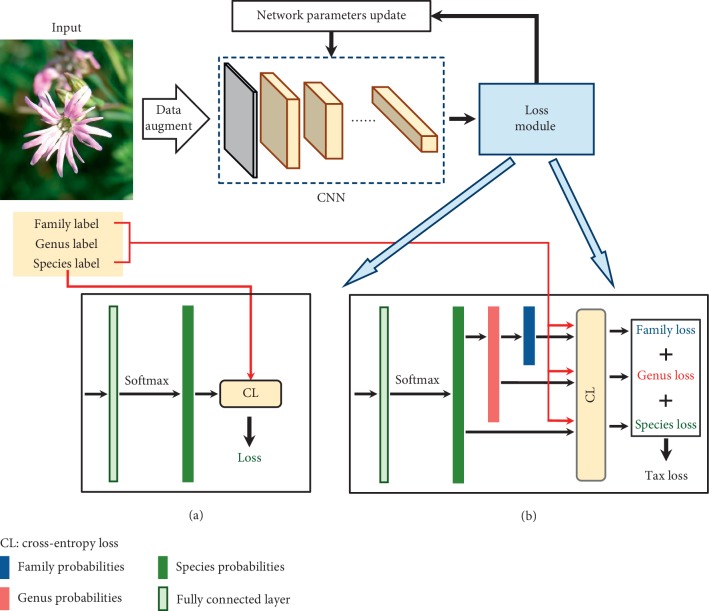
The end-to-end training pipeline of deep learning plant identification. Two distinct loss modules are shown in the lower part: (a) the cross-entropy loss uses only the species-level labels; (b) the proposed taxonomic loss encodes the hierarchy among three-level labels into the objective function.

Figure 1 shows the end-to-end training pipeline of deep learning plant identification with two different loss modules. First, each image was augmented randomly and resized to a fixed resolution and then fed into the convolutional neural network (CNN) to extract high-dimensional features by multiple layers abstraction. Next, the loss module was applied for comparative analysis between the CNN predictions and ground truth. Finally, the network parameters were updated by the optimizer according to the loss value.

The most adopted loss module [22] is shown in Figure 1(a), which generated loss only based on one-level label, usually species-level label. The CNN output was connected to a fully connected (FC) layer with *n* neurons to produce *n*-bit species logits where *n* was the number of species. After the calculation of softmax function, the *n*-bit species logits were converted into *n*-bit species probabilities. Then, the cross-entropy loss function was designed to measure the performance of multiclass classification with one-level labels, and it was calculated between the species probabilities and species-level label as follows:

(1) $$\begin{array}{c} l_{s-\text{CE}}=-\overset{n}{\underset{i=1}{\sum }}t_{i}\log\left(p_{i}\right), \end{array}$$

and *p*_*i*_ is calculated by softmax function as

(2) $$\begin{array}{c} p_{i}=\frac{e^{z_{i}}}{\sum_{k=1}^{n}e^{z_{k}}}, \end{array}$$

where *n* represents the number of species, [*z*_1_, *z*_2_,…, *z*_*n*_] represents the FC layer output, and the one-hot code of species-level label is [*t*_1_, *t*_2_,…, *t*_*n*_]. In this way, although the model made the finest-grained species-level predictions, the coarser-level predictions could only be backward inferred along the taxonomic tree, which ignored the supervision information of coarser-level labels.

### 3.1. Taxonomic Loss

In order to fully exploit multilevel labels and hierarchical relationships among them, the taxonomic loss was proposed. As illustrated in Figure 1(b), softmax function was applied on the output of FC layer to generate *n*-bit species probabilities. Later, the species probabilities were progressively transformed to genus and family probabilities according to the taxonomic tree. Then three-level cross-entropy losses were calculated, respectively, between the label and probabilities at corresponding level. Finally, the taxonomic loss was the sum of all three-level losses and used in the following optimization algorithm for network parameters updating.

**Figure 2:**
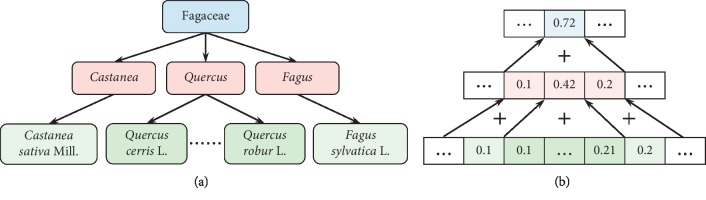
(a) A brief taxonomic tree of Fagaceae family; (b) the derivation process of genus probabilities and family probabilities according to the taxonomic hierarchy by group and sum operation.

The key to calculating taxonomic loss was converting the species probabilities into genus probabilities and family probabilities according to taxonomic tree. The species probabilities were the output of CNN after softmax normalization. Firstly, each bit of species probabilities belonging to same genus were grouped and then summed to generate one bit on genus level. After all species bits were grouped and summed, the *m*-bit genus probabilities were derived, where *m* is the number of classes on genus level. Secondly, all bits of the derived genus probabilities were further grouped and summed according to the family-genus hierarchy to generate the family probabilities. In this way, the genus probabilities and family probabilities were progressively derived from the species probabilities according to the taxonomic tree. A sample progressive derivation of high-level probabilities is illustrated in Figure 2(b), which is corresponding to the taxonomic tree shown in Figure 2(a). As shown in Figure 2, the *Quercus cerris* L. bit, *Quercus robur* L. bit, and other species-level bits belonging to *Quercus* are grouped together and the values of them are summed to get the probability of *Quercus* at genus level. Next, the value of *Quercus* bit is further added to the *Castanea* bit and *Fagus* bit to generate the Fagaceae bit at family level, which is equal to 0.72. Specifically, the genus probabilities and family probabilities are calculated as follows:

(3) $$\begin{array}{c} \begin{array}{cc} g_{j}=\underset{k}{\sum }s_{k}, & \text{where }S\left[k\right]\text{ belongs to }G\left[j\right], \end{array}\\ \begin{array}{cc} f_{i}=\underset{j}{\sum }g_{j}, & \text{where }G\left[j\right]\text{ belongs to }F\left[i\right], \end{array} \end{array}$$

where *f*/*g*/*s*_*x*_ is the value of family/genus/species probability at *x*-th bit and *F*/*G*/*S*[*x*] is the *x*-th family, genus, or species.

After multilevel probabilities were generated by the CNN softmax output and the following group and sum operation along taxonomic tree, the cross-entropy of each level, *l*_*f–*CE_, *l*_*g*−CE_, *l*_*s–*CE_, were calculated independently between the predicted probabilities and ground truth by equation (1). Finally, the taxonomic loss was the sum of the multilevel cross-entropy losses as follows:

(4) $$\begin{array}{c} l_{\text{TAX}}=l_{f-\text{CE}}+l_{g-\text{CE}}+l_{s-\text{CE}}. \end{array}$$

The group and sum operation encoded the taxonomic tree into the deep learning objective function, which was easy to implement and compatible with end-to-end training. Also, when misclassification happened at coarse granularity, the taxonomic loss could provide more information than cross-entropy loss. Due to the use of taxonomic loss, more supervision information could be leveraged to improve the performance of plant identification models.

### 3.2. End-To-End Training

**Figure 3:**
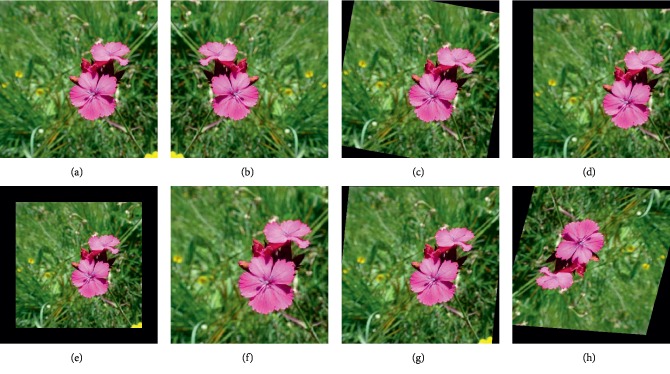
The effects of image augmentation. (a) The cropped image, the image with (b) horizontal flipping, (c) rotation (degree = 10), (d) translation (ratio = 0.15), (e) scaling (ratio = 0.8), (f) scaling (ratio = 1.2), (g) shear (degree = 10), and (h) multiple random augmentation.

The experiments were implemented by Pytorch deep learning framework. The CNNs were trained end-to-end on a workstation with one Nvidia GeForce GTX Titan Xp GPU (12 GB graphic memory). All the models loaded ImageNet pretrained weights for initialization and were trained over 100 epochs. The basic learning rate was 0.01, and it was dropped by half after every 30 epochs. The stochastic gradient descent (SGD) with 90% momentum was used to optimize the network parameters. All of the methods were compared on test sets of PlantCLEF 2015 and PlantCLEF2017 dataset. Besides, to improve the robustness of model, data augmentation was applied in the experiments. Each image was center cropped, and the images were resized to 299 × 299 pixels when the Inception-v3 and Inception-ResNet-v2 were adopted for feature extracting, and the images were resized to 224 × 224 pixels when using the other CNNs. Finally, all the cropped images were handled by several processing methods: flipping, rotation, translation, scaling, and shear.  Figure 3 shows the effects of data augmentation in the experiments.

## 4. Results

### 4.1. Results on PlantCLEF 2015 Dataset

Several state-of-the-art neural networks were trained, respectively, by two loss functions shown in Figure 1: the commonly used cross-entropy loss and the proposed taxonomic loss. The experimental results of different models in the testing set are depicted in Table 2. In addition to the most frequently used species accuracy for algorithm evaluation, the genus accuracy and family accuracy were also taken into account. As seen from Table 2, the models trained by taxonomic loss are consistently better in performance than those trained by cross-entropy loss, and the improvements of species accuracy range from 0.08% to 2.45%. The SENet-154 trained by taxonomic loss outperforms the other models, which achieves family, genus, and species accuracies of 83.19%, 78.08%, and 71.15%, respectively. Meanwhile, the Inception-ResNet-v2 trained by taxonomic loss obtains the most significant performance increase compared with the cross-entropy one and improves three-level accuracies of 2.70%, 2.28%, and 2.45%. These experimental results demonstrated that the proposed taxonomic loss was easy to implement and could effectively facilitate the training of both light-weight and complex neural networks.

**Figure 4:**
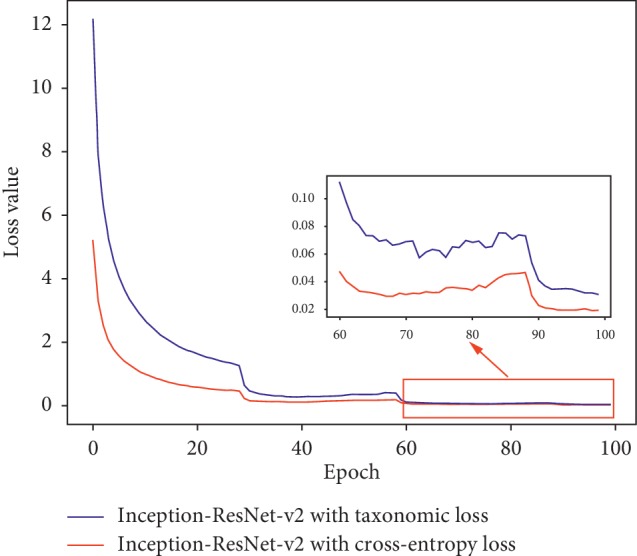
Loss curves of Inception-ResNet-v2 trained by cross-entropy and taxonomic loss.

Figure 4 illustrates the loss descent curves of Inception-ResNet-v2 trained by two different loss functions during the training stage. It can be seen that the value of taxonomic loss is much larger than cross-entropy loss at the beginning stage, because it is the sum of three-level losses. As training advancing, the difference between them was gradually decreasing. Although the taxonomic loss value was slightly higher than the cross-entropy one at the final stage, the decline was greater and the optimization for network was also better.

### 4.2. Results on PlantCLEF 2017 Dataset

In the latter experiments, the state-of-the-art CNNs were trained by cross-entropy and taxonomic loss on PlantCLEF 2017 dataset to further verify the proposed algorithm. As shown in Table 3, when the neural networks trained by the taxonomic loss, almost all of them deliver greater than 2% family accuracy improvements, and the species accuracy increase range from 0.50% to 3.18%. The SENet-154 trained by taxonomic loss performs better than the others, which achieves three-level accuracies of 84.07%, 79.97%, and 73.61% and obtains 2.23%, 1.34%, and 1.08% relative improvements compared with the same model trained by cross-entropy loss. Therefore, it can be concluded that the taxonomic loss could also further facilitate the training of various neural networks on PlantCLEF 2017 dataset with huger data and more species.

Also, the proposed taxonomic loss could generate more supervision information when coarse-level predictions were wrong, which improved the accuracies of family and genus levels. Several typical plant images in PlantCLEF 2017 testing set and their corresponding predictions are listed in Table 4. One can see that the ResNet-50 trained by cross-entropy loss identified all images incorrectly at three levels, while the model trained by the taxonomic loss could correct the predictions at coarse levels. For example, the sample (b) was recognized as *Fagus grandifolia* Ehrh. at species level by the ResNet-50 trained by cross-entropy loss, and the coarser-level labels (Fagus, *Fagaceae*) were inferred according to the taxonomic tree, so the three-level predictions were totally wrong. Although the model trained by the proposed taxonomic loss had not predicted the most specific species correctly, the family and genus were correct, which was also useful in practice.

## 5. Discussion

Based on the above results, it has been verified that the proposed taxonomic loss could further facilitate the training of multiple state-of-the-art neural networks no matter on PlantCLEF 2015 dataset with 1,000 species or PlantCLEF 2017 dataset with 10,000 species. To further validating the influence of taxonomic tree structure on model optimization, the compared experiments were conducted. As shown in Table 5, two neural networks were additionally trained by two-level taxonomic loss: the family-species structure (F-S) and the genus-species structure (G-S), while “F-G-S” represents the taxonomic loss shown in Figure 1(b) and “S” indicates the cross-entropy loss shown in Figure 1(a). One can see from Table 5 that the models trained by three-level taxonomic loss consistently outperform the two-level ones, and both of them achieve higher accuracies than the models trained by single-level taxonomic loss, also known as cross-entropy loss. These experimental results have demonstrated that the taxonomic hierarchy with more levels could provide more supervision information during the training stage of neural networks and achieve more competitive results.

## 6. Conclusion

In this paper, a loss function for fine-grained plant image identification was proposed, which could encode the hierarchical relationships of taxonomic tree into the deep learning objective function. On the one hand, the proposed method was easy to implement with simple group and sum operation. And on the other hand, it facilitated the end-to-end training of various neural networks, which further increased plant identification accuracies at species, genus, and family levels. The experiments on PlantCLEF 2015 and PlantCLEF 2017 datasets demonstrated that the proposed taxonomic loss function performed better than the most adopted cross-entropy loss. In the future, the taxonomic loss could be generalized to other fine-grained classification tasks with multilevel labels, such as bird species identification and car class categorization.

## Figures and Tables

**Table 1:** Details of PlantCLEF 2015 and PlantCLEF 2017 dataset.

Dataset	Number of classes			Number of samples	
	Family	Genus	Species	Train	Test
PlantCLEF 2015	124	516	1,000	91,758	21,446
PlantCLEF 2017	341	2,991	10,000	226,386	29,901

**Table 2:** Accuracies of eight state-of-the-art neural networks trained by cross-entropy and taxonomic loss on PlantCLEF 2015 dataset.

Neural network	Loss function	Accuracy (%)		
		Family	Genus	Species
GoogLeNet [13]	CL	72.62	65.97	59.69
	TAX	**74.95**	**68.07**	**61.06**

ResNet-50 [23]	CL	77.48	71.59	65.07
	TAX	**78.55**	**72.20**	**65.15**

Inception-v3 [14]	CL	77.93	74.01	67.98
	TAX	80.31	**74.46**	67.42

Inception-ResNet-v2 [11]	CL	80.66	**74.57**	67.93
	TAX	**83.36**	76.85	**70.38**

MobileNet v2 [24]	CL	72.13	65.65	59.16
	TAX	**74.18**	**67.52**	**60.42**

ShuffleNet v2 [25]	CL	66.39	59.32	52.88
	TAX	**68.80**	**61.45**	**54.18**

DenSeNet-169 [26]	CL	78.57	73.00	66.76
	TAX	**80.11**	**74.48**	**67.23**

SENet-154 [27]	CL	81.25	76.81	70.08
	TAX	**83.19**	**78.08**	**71.15**

**Table 3:** Accuracies of eight state-of-the-art neural networks trained by cross-entropy and taxonomic loss on PlantCLEF 2017 dataset.

Neural network	Loss function	Accuracy (%)		
		Family	Genus	Species
GoogLeNet [13]	CL	68.73	64.22	57.86
	TAX	**73.29**	**68.64**	**61.04**

ResNet-50 [23]	CL	76.32	72.49	66.68
	TAX	**78.95**	**74.77**	**68.04**

Inception-v3 [14]	CL	77.32	73.12	67.05
	TAX	**79.87**	**76.02**	**68.76**

Inception-ResNet-v2 [11]	CL	79.98	75.43	68.97
	TAX	**82.31**	**78.65**	**71.21**

MobileNet-v2 [24]	CL	71.76	67.73	61.78
	TAX	**73.88**	**69.40**	**62.01**

ShuffleNet-v2 [25]	CL	61.94	57.12	49.96
	TAX	**66.13**	**60.73**	**53.12**

DenSeNet-169 [26]	CL	76.34	72.53	66.60
	TAX	**77.87**	**73.92**	**67.10**

SENet-154 [27]	CL	81.84	78.63	72.53
	TAX	**84.07**	**79.97**	**73.61**

**Table 4:** Typical images in PlantCLEF 2017 testing set and predictions made by ResNet-50 trained by two different loss functions: cross-entropy loss (CL) and taxonomic loss (TAX).

		Loss function	Family	Genus	Species
a	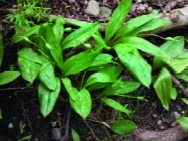	GT	**Lilliaceae**	***Erythronium***	***Erythronium americanum* Ker Gawl.**
		CL	Orchidaceae	*Orchis*	*Orchis mascula* (L.) L.
		TAX	**Lilliaceae**	*Clintonia*	*Clintonia andrewsiana* Torr.

b	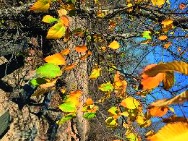	GT	**Ulmaceae**	***Ulmus***	***Ulmus americana* L.**
		CL	Fagaceae	*Fagus*	*Fagus grandifolia* Ehrh.
		TAX	**Ulmaceae**	***Ulmus***	*Ulmus crassifolia* Nutt.

c	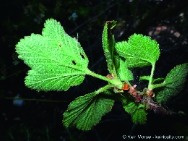	GT	**Grossulariaceae**	***Ribes***	***Ribes indecorum* Eastw.**
		CL	Rosaceae	*Holodiscus*	*Holodiscus discolor* (pursh) Maxim.
		TAX	**Grossulariaceae**	***Ribes***	***Ribes indecorum* Eastw.**

d	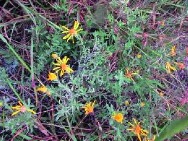	GT	**Asteraceae**	***Heterotheca***	***Heterotheca canescens* (DC.) Shinners**
		CL	Leguminosae	*Syrmatium*	*Syrmatium glabrum* Vogel.
		TAX	**Asteraceae**	***Heterotheca***	***Heterotheca canescens* (DC.) Shinners**

Bold values indicate the ground truth (GT) and the correct predictions.

**Table 5:** Accuracies of two neural networks trained by taxonomic loss with different taxonomic hierarchy.

Dataset	Neural network	Taxonomic hierarchy	Accuracy (%)		
			Family	Genus	Species
PlantCLEF 2015	Inception-ResNet-v2 [11]	F-G-S	83.36	76.85	70.38
		F-S	82.04	76.10	69.36
		G-S	81.48	75.82	68.94
		S	80.66	74.57	67.93

PlantCLEF 2017	ShuffleNet-v2 [25]	F-G-S	66.13	60.73	53.12
		F-S	64.08	57.71	50.03
		G-S	64.26	59.11	51.64
		S	61.94	57.12	49.96

## Data Availability

The PlantCLEF 2015 dataset and PlantCLEF 2017 dataset supporting this study are from previously reported studies, which have been cited. The PlantCLEF 2015 dataset is available at http://otmedia.lirmm.fr/LifeCLEF/PlantCLEF2015/, and the PlantCLEF 2017 dataset is available at http://otmedia.lirmm.fr/LifeCLEF/PlantCLEF2017/
